# Lectures on the Theory and Practice of Physic

**Published:** 1848-08

**Authors:** 


*Lectures on the Theory and Practice of Physic.* By JOHN BELL, M. D., etc., etc., and WILLIAM STOKES, M. D. etc., etc. Fourth edition, revised and enlarged. In two volumes, 8vo. Ed. Barrington and Geo. D. Haswell. Philadelphia, 1848.

These ponderous volumes contain seventeen hundred and sixty pages, of which more than fourteen hundred are from the pen of our learned and industrious countryman. Dr. Bell has the gratification of knowing, from the rapid sale of the former edition, that his great labour has been rightly appreciated by the profession, and that BELL AND STOKES'S LECTURES now rank among the standard works of the day.

In the Preface we are informed that the present edition has been revised with considerable care, and many of the subjects—such as *Epidemic Cholera*, *Diseases of the Urinary Organs*, *Diseases of the Female Organs of Generation*, *Pulmonary Consumption*, *Diseases of the Heart*, *Meningitis* (*Simple and Tubercular*), the *Exanthemata*, and *Fevers*—have been recast. Others are introduced for the first first time, viz., *Diseases of the Eye*, *Diseases of the Bloodvessels*, and *Dropsy*.

We cannot pretend to examine the Lectures on these various subjects in detail, but it would be wrong to pass over some of them without a brief notice.

The whole subject relating to epidemic cholera, which, from its threatening progress in the north of Europe, is now occupying a large space in European journals, is thoroughly investigated. Dr. Bell is a believer in some peculiar atmospheric change or condition as a general predisposing cause, as in the case of influenza, catarrh, &c., and an utter unbeliever in its contagious nature.

"The shortness of duration of epidemic cholera in a place, the suddenness and rapid diffusion of the disease beyond what could occur from personal intercourse, and its entire disappearance, are facts adverse to a belief that it is contagious. To the same purport is the disorder of the digestive organs among the inhabitants, preceding its regular attack, and anterior, also, to any imputed importation or intercourse of any kind with the sick in other parts."

Much attention is given to diseases of the respiratory organs, and especially to phthisis. The nature, causes, and formation of tubercle, in the lungs and various other parts of the body, is full and complete; indeed, we know of no better treatise on the subject than is contained in these Lectures. In the Lectures on *Diseases of the Eye*, *Diseases of the Bloodvessels*, and *Dropsy*, which are contained in this edition, but not in those which preceded, we have observed nothing special to remark. The subjects are treated in the same clear and satisfactory manner observable on other topics, on which the author's personal experience enables him to speak with more authority.

As might be expected, the great object of giving a true account of our present knowledge on the various subjects treated of, instead of affected originality, is conspicuous throughout. To use the author's own language: "In the performance of his task, the American contributor has not been backward in adducing his own experience, or in the independent expression of his own matured convictions; but, at the same time, he disclaims the particular absurdity of professing to write an original work on the practice of medicine. When the materials are in such large proportion common to all, a self-attribution of originality may well create suspicion of the want of good sense, if not of good faith, of the claimant. As well might we look for an original cyclopædia, or an original dictionary. How small, in the nature of things, is the amount of positive knowledge, verified by actual observation and experiment, peculiar to any single inquirer, however industrious and conscientious he may be. Still smaller must be the proportion of real discovery, originating with himself. He, therefore, who would obtain credit for an original work on the pathology and treatment of the several diseases included in the nosological catalogue, or even the chief of them, written with an approach to the minuteness of detail met with in the systematic treatises extant, must either draw largely from his imagination or be inspired. He must write a romance, or be favoured with a special revelation."

No well informed man can doubt the correctness of these remarks, or the justness of the rebuke which they administer to that small fry of critics who affect to despise every thing that does not pretend to originality. With such, the labour and judgment employed in collecting the materials which lie scattered and useless, and the genius and knowledge required to combine and arrange them for useful purposes, is nothing. The architect who constructs the convenient edifice is a useless being in comparison with him who digs the sand or quarries the stone. The jewel of philosophy is due to him who first *saw* the apple fall, and not to the mind that deduced from that fact the great physical law! From critics of this description, Dr. Bell need expect nothing but abuse. He will be denounced as "*a compiler*"—"*a book worm*"—"not original"—"not a practical man"—by some who will forget, if not ignorant of, the truths conveyed in the remarks we have quoted. He may expect, indeed, whenever the occasion presents, to find himself branded as we have mentioned, or perhaps stigmatized as a plagiarist, and long extracts from his volumes detailing the symptoms of intermittent fever, or pleurisy, or jaundice, with corresponding passages from Louis, or Cullen, or, it may be, Hippocrates, torn from their proper connection and made to stare most knowingly at each other in parallel columns! To such reviewers we commend a further extract from our author's Preface.

"Some writers deceive themselves, if not their readers, into a belief that, if they use their own language, they give a stamp of originality to ideas derived from the common stock. They forget, that a temporary control of the mint and the imprint of their effigy on the coin, are not the circumstances on which depend its intrinsic value and currency. A desirable homogeneousness of appearance is obtained by a certain unity of style; but it may still be questioned, whether a description of disease is less vivid in the very words of Cullen, or a matter of fact is less tersely expressed in those of Heberden or Louis, than if the description and fact were conveyed in the choicest phrases of the lecturer or writer at the time."

